# The Potential Public Health Impact of the Adjuvanted Respiratory Syncytial Virus Prefusion F Protein Vaccine Among Older Adults in Italy

**DOI:** 10.3390/vaccines13030212

**Published:** 2025-02-20

**Authors:** Anna Puggina, Filippo Rumi, Eleftherios Zarkadoulas, Alen Marijam, Giovanna Elisa Calabró

**Affiliations:** 1 GSK, Verona, Italy; 2 Graduate School of Health Economics and Management (ALTEMS), Università Cattolica del Sacro Cuore, Rome, Italy; filippo.rumi@unicatt.it; 3 GSK, Wavre, Belgium; eleftherios.x.zarkadoulas@gsk.com (E.Z.); alen.x.marijam@gsk.com (A.M.); 4 Value in Health Technology and Academy for Leadership and Innovation (VIHTALI), Spin-Off of Università Cattolica del Sacro Cuore, Rome, Italy; alisacalabro@icloud.com; 5 Department of Human Sciences, Society and Health, University of Cassino and Southern Lazio, Cassino, Italy

**Keywords:** Italy, public health impact, older adults, respiratory syncytial virus, vaccine

## Abstract

**Background:** Respiratory syncytial virus (RSV) is a common cause of acute respiratory infection (ARI). The risk of severe RSV outcomes is higher among older adults (OAs) and individuals with chronic diseases (high risk, HR). AS01_E_-adjuvanted RSV preFusion protein 3 OA vaccine (adjuvanted RSVPreF3 OA is approved for the prevention of lower respiratory tract disease [LRTD] due to RSV in OAs). The objective of this study was to assess the potential public health impact of an RSV vaccination program using adjuvanted RSVPreF3 OA in adults ≥75 years (y) and HR adults ≥60 y in Italy. **Methods:** A static multi-cohort Markov model was used to estimate the number of RSV cases and associated health outcomes projected in adults ≥75 y and HR adults ≥60 y with no RSV vaccination or with a single dose of adjuvanted RSVPreF3 OA. Epidemiological, healthcare resource use and cost data were obtained from the scientific literature. Vaccine efficacy and waning inputs were based on results from the AReSVi-006 phase III clinical trial. Several scenarios for vaccine coverage were explored. **Results:** Assuming the target vaccination rate for influenza vaccination in Italy (75%), the model predicted that vaccinating Italian adults ≥75 y and the HR population ≥ 60 y with adjuvanted RSVPreF3 OA would reduce the number of RSV-LRTD events by 43%, leading to a reduction in associated emergency department visits, hospitalizations, complications, deaths, and direct healthcare costs over a 3-year period. **Conclusions:** The vaccination of Italians aged ≥ 75 y and HR individuals aged ≥ 60 y using the adjuvanted RSVPreF3 OA vaccine has the potential to offer substantial public health benefits by reducing the burden of RSV disease.

## 1. Introduction

Respiratory syncytial virus (RSV) is one of the most frequent causes of acute respiratory infections in adults [1]. Most of the children are infected with RSV as infants or toddlers before their second birthday, but repeat infections can occur throughout life [2]. RSV is transmitted by coughing or sneezing, and infected individuals are typically contagious for 3 to 8 days, although some infants and individuals with compromised immune systems may remain contagious for several weeks, even after symptoms have resolved [2]. RSV circulation is highly seasonal, occurring mainly in the winter in temperate climates [1]. In Italy, RSV is active between November and April, ref. [3] with a peak in hospitalizations from December to February [4].

The clinical features of RSV are not generally different from other respiratory viruses and vary from asymptomatic carriage to acute respiratory distress [1]. Upper respiratory symptoms include nasal congestion, runny nose, and sore throat a few days after infection [1]. If the infection progresses to the lower respiratory tract, symptoms such as cough, wheezing, and dyspnea may follow [1]. In the lower respiratory tract, RSV infection can be associated with severe respiratory complications such as pneumonia, bronchitis, and the exacerbation of asthma or chronic obstructive pulmonary disease (COPD) [5,6,7]. Furthermore, cardiovascular complications can also follow an RSV lower respiratory tract infection, with problems such as myocardial infarction, stroke, and heart failure [8,9]. Older adults (OA), individuals with chronic illnesses, transplant patients, and patients with COPD are among the groups most at risk of developing severe disease, which may result in prolonged hospitalization, the need for intensive care, and death [10,11,12].

The disease burden attributable to RSV in OA is substantial. In the United States (US), a prospective study estimated that RSV developed annually in 3–7% of healthy adults aged 65 years (y) or more and in 4–10% of high-risk adults (individuals with chronic heart or lung disease) and accounted for 10.6% of hospitalizations for pneumonia, 11.4% for COPD, 7.2% for asthma, and 5.4% for congestive heart failure [13]. In the first systematic review and meta-analysis investigating RSV disease burden in Italy, the positivity prevalence for RSV was higher in OAs (4.4%) than in working-age adults (3.5%). RSV prevalence was also higher in outpatient (4.9%) than inpatient (2.9%) settings. Furthermore, in-hospital mortality was estimated to be as high as 7.2% [14]. Based on an international systematic literature review and network meta-analysis conducted in 2023, every year in Italy, there are roughly 290,000 cases of RSV-ARI, 26,000 hospitalizations, and 1800 in-hospital deaths in adults aged ≥ 60 y [15]. In Italy, RSV is associated with a considerable clinical and economic burden [16]. A retrospective database study in Italy found that in patients aged 60 y or over and hospitalized with RSV, the mean direct healthcare cost per patient was EUR 11,599, of which 78.9% related to hospitalization, 16.2% to prescription medications, and 4.9% to outpatient services [17].

However, there are indications that current estimates may not reflect the true clinical and economic burden of RSV, and diagnostic testing characteristics (such as variations in clinical samples and diagnostic tools) may lead to a wrong assessment of disease burden [18]. For example, only half of the cases confirmed by reverse transcriptase polymerase chain reaction also showed a positive viral culture, and the use of nasal swabs alone may underestimate RSV disease rates compared with adding the testing of sputum or oropharyngeal samples [18]. Surveillance systems based on influenza-like illness or fever may under-detect RSV in adult outpatients, as fever in RSV-positive cases is not a common symptom [19]. Using acute respiratory illness (ARI) with cough as the RSV case definition may improve specificity [19]. It has been estimated that the true RSV-associated hospitalization burden may be 2.2 times higher than reported [18].

Currently, no RSV disease-specific treatment has been approved for adults aged 60 y or more. The GSK AS01_E_-adjuvanted RSV preFusion protein 3 OA (adjuvanted RSVPreF3 OA) vaccine has shown efficacy in preventing RSV infection and RSV-associated lower respiratory tract disease (LRTD) in adults aged 60 y or more [20]. AS01_E_ is an adjuvant system containing 3-*O*-desacyl-4′-monophosphoryl lipid A (MPL), QS-21—a saponin extracted from the bark of the *Quillaja saponaria* Molina tree—and liposome (25 µg of MPL and 25 µg of QS-21). Vaccine efficacy in the first season was 82.6% against RSV-LRTD overall, 71.7% against RSV-ARI overall, 93.8% against RSV-LRTD in participants aged 70–79 y, and 94.6% against RSV-LRTD in participants aged 60 y or more with at least one comorbidity [20]. The vaccine was well tolerated and had an acceptable safety profile [20]. Follow-up data over two RSV seasons indicated that protection was durable, with vaccine efficacy after a single dose of adjuvanted RSVPreF3 OA over two seasons (median efficacy follow-up of 17.8 months) reported at 67.2% against RSV-LRTD and 78.8% against severe RSV-LRTD. The corresponding values for 13.9 months of follow-up were 77.3% and 84.6%, respectively [21]. The adjuvanted RSVPreF3 OA vaccine became the world’s first vaccine approved for the prevention of LRTD due to RSV in adults aged 60 y or over in the US in May 2023 [22] and in the European Union in June 2023 [23]. The Italian Ministry of Health has issued a document on preventive and immunization measures for RSV, recognizing RSV’s impact on OAs and individuals with chronic illnesses, and listing the available preventive and immunization interventions currently authorized in Italy, including the adjuvanted RSVPreF3 OA vaccine [24]. The Vaccination Calendar for Life is an Italian alliance of scientific and professional societies of public health physicians, pediatricians, and general practitioners, whose aim is to provide a periodical update on the optimal, scientifically driven vaccination calendar throughout life [25]. It currently recommends RSV vaccination for use in individuals aged ≥75 y, and those aged 60 y or over with chronic diseases (high risk, ≥60 y HR) [26]. In February 2024, the Hygiene and Infectious Disease Societies also recommended that including RSV vaccination in adults aged > 75 y and adults aged > 60 y with comorbidities should be considered [27].

**Figure 1:**
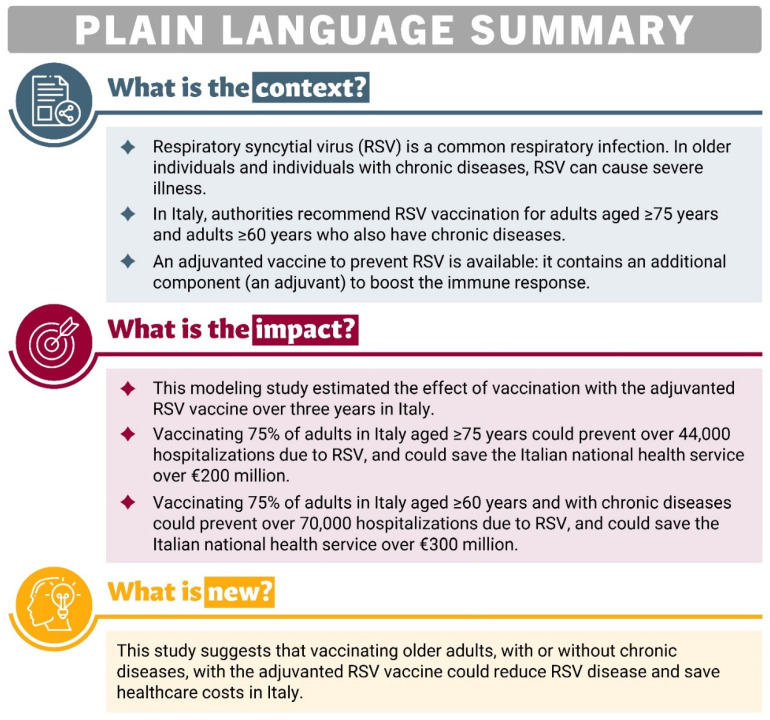
Plain-language summary.

Preventive interventions such as RSV vaccination in adults at high risk and those aged 75 y or over could offer not only potential clinical benefits by reducing disease burden but also possible economic benefits by reducing healthcare and social costs. Recent modeling analyses have indicated that the adjuvanted RSVPreF3 OA vaccine could considerably reduce RSV disease burden in adults 60 y or more across 11 European countries, as well as in adults 65 y or older in Italy, in adults 60 y or older in the US, and adults aged 60 y or more in Japan [16,28,29,30]. The objective of the present study was to assess the public health impact (e.g., reductions in RSV-related cases, hospitalizations, and other outcomes) of a vaccination program using the adjuvanted RSVPreF3 OA vaccine in the two populations in which its use is currently recommended in Italy, the population aged ≥75 y and the HR population aged ≥60 y, over a 3-year time horizon (corresponding to three consecutive RSV seasons). A plain-language summary of this study is provided in Figure 1.

## 2. Methods

### 2.1. Adult Respiratory Syncytial Virus Cost-Effectiveness Model (ARIEL)

**Figure 2:**
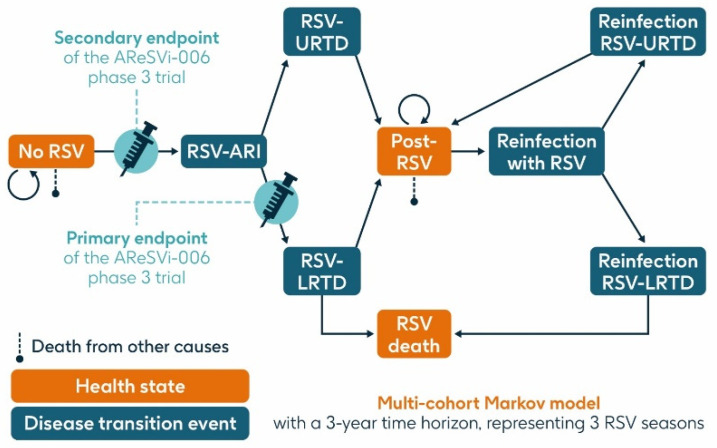
Model structure (adapted from Kurai et al. 2024 [30]). ARI, acute respiratory infection; LRTD, lower respiratory tract disease; RSV, respiratory syncytial virus; URTD, upper respiratory tract disease.

A previously published static multi-cohort Markov model [16,28,29,30] was used to estimate the projected number of RSV cases and related health outcomes over a 3-year time horizon in the population aged ≥75 y and the HR population aged ≥60 y in Italy without RSV vaccination or with a vaccination program using a single dose of the adjuvanted RSVPreF3 OA vaccine at varying levels of coverage. The model structure is summarized in Figure 2.

The model structure and data inputs of this model were validated by two Italian experts in epidemiology and public health (GEC) and health economics (FR).

The model had three health states: ‘no RSV’, ‘post-RSV’ (individuals who have recovered from RSV infection), and ‘RSV death’. It included three transition events: RSV-ARI, RSV-URTD (upper respiratory tract disease associated with RSV), and RSV-LRTD, each of which could occur as either a first infection within the timeframe of the model or as a reinfection in subsequent seasons, since infection with RSV does not result in persistent immunity. Reinfection within one year was not included.

All individuals entered the model in the ‘no RSV’ state in which they could remain, or they could transition through the RSV-ARI transition event (first RSV infection experienced by a given individual within the model) and then either through the RSV-URTD or RSV-LRTD transition events. Individuals transitioning through the RSV-URTD transition event entered the ‘post-RSV’ health state, and those transitioning through the RSV-LRTD transition event could either enter the ‘RSV death’ or ‘post-RSV’ health state. Only the RSV-LRTD transition event could result in RSV death. As this is a static model, subjects cannot progress from the URTD to the LRTD transition event, whereas in the real world, URTD cases can progress to LRTD and vice versa. This may result in an underestimation of RSV-related events in our model. Individuals in the ‘post-RSV’ health state could experience reinfection with RSV and associated LRTD and URTD transition events in a similar way to the first infection. Those in the ‘no RSV’ and ‘post-RSV’ health states could also die from causes unrelated to RSV, based on general population mortality.

The cycle time of the model was one month, reflecting the acute nature of RSV infection.

Costs and healthcare resource use were assigned to the RSV-URTD and RSV-LRTD transition events and tracked throughout the model.

### 2.2. Main Assumptions

The model compared a single dose of the adjuvanted RSVPreF3 OA vaccine with a strategy of no vaccination, as there is currently no existing RSV vaccination program in Italy. The time horizon was three years, corresponding to three consecutive RSV seasons. This was based on data from the AReSVi-006 phase III clinical trial [21], showing that one dose of the adjuvanted RSVPreF3 OA vaccine provided protection for two full seasons, and regression analysis indicating substantial residual protection during the third season [29,30]. Costs and health outcomes were discounted at 3.0% per year, in line with Italian Medicines Agency (AIFA) guidelines [31].

It was assumed that in this analysis, the adjuvanted RSVPreF3 OA vaccine was administered in October, as this is typically the start of the RSV season in Italy. No data are available on revaccination with the adjuvanted RSVPreF3 OA vaccine, and therefore, revaccination was not considered in the analysis.

It was assumed that individuals could not experience RSV reinfection during the same season, but that they would become susceptible to infection again in subsequent seasons. It was assumed that RSV-URTD cases incurred no additional mortality risk, and that RSV-related mortality occurred only in RSV-LRTD cases. To maintain a conservative approach, it was also assumed that RSV-URTD cases would require no medical attention and therefore would incur no healthcare resource use or associated costs.

### 2.3. Model Inputs

A targeted literature review was performed to identify data on RSV epidemiology, healthcare resource utilization, and costs at an international level with a focus on the Italian context, which highlighted the scarcity of Italian specific data. When no country-specific data could be found, data from European studies or from similar countries were used and were selected using a conservative approach.

#### 2.3.1. Demographic and Coverage Data—Population Aged ≥75 y

The size of the general Italian population stratified by age was obtained from the Istituto Nazionale di Statistica (Istat) [32], and data are shown in Supplementary Table S1.

Several scenarios with different vaccine coverage rates were investigated in the model, all derived from influenza vaccination rates. Influenza vaccination was used as a proxy, since the adjuvanted RSVPreF3 OA vaccine is expected to be administered annually alongside the seasonal influenza vaccination in Italy. In individuals aged ≥75 y, the scenarios for vaccine coverage were as follows:

56.7%, which is the influenza vaccination rate in the 2022–2023 season in adults aged 65 y or older [33];

75%, which is the minimum target for influenza vaccination in Italy [34];

95%, which is the optimum target for influenza vaccination in Italy [34].

#### 2.3.2. Epidemiology Data—Population Aged ≥75 y

The incidence of RSV-ARI was based on that of medically attended RSV in OAs reported in a European study [35] and assumed to be the same in all age groups (as the data were not stratified by age). The annual incidence of RSV infection in the model was distributed across the months based on the reported RSV cases per month in the Lombardy region of Italy during the 2022–2023 influenza season [36] (Supplementary Table S2).

The proportion of RSV-ARI cases that were RSV-LRTD cases was set at 47.6%, based on data from a phase III clinical trial [20], and the remaining 52.4% were assumed to be RSV-URTD cases (Supplementary Table S2).

The age-adjusted probabilities of developing complications of pneumonia, asthma, or COPD associated with an RSV-LRTD event were obtained from a retrospective study conducted in Southern Europe by Boattini et al. [37].

All-cause general population mortality was based on Italian life tables by age obtained from Istat [38]. No country-specific data were available on mortality rates for RSV-LRTD in Italy, so data from the Statens Serum Institute in Denmark, reporting the probability of death in patients hospitalized with RSV-LRTD, were used as a proxy [39] (Supplementary Table S2). Danish data from the 65–74 age group were also used for the 60–64 age group (data not available for the latter age group). The RSV-LRTD hospitalization rate reported by Belongia et al. [40] was then used and multiplied by the in-hospital mortality rate from the Danish data to obtain the probability of death from RSV-LRTD.

#### 2.3.3. Healthcare Resource Utilization and Cost Data—Population Aged ≥75 y

The healthcare resources considered in the model were outpatient visits, hospitalizations, emergency department visits, intensive care visits, and antibiotic use (Supplementary Table S3). To maintain a conservative approach, it was assumed in this analysis that none of the RSV-URTD cases required medical attention and therefore incurred no healthcare resource costs.

In the study on RSV incidence [35], 31.0% of all RSV-ARI cases were medically attended, and it was assumed that all these medically attended cases were RSV-LRTD cases. Of the RSV-ARI cases in the model, 47.6% were RSV-LRTD cases (as described above), and, therefore, the percentage of RSV-LRTD cases that were medically attended was set at 65.13%. All medically attended RSV-LRTD cases were assumed to require one outpatient visit. The proportion of RSV-LRTD cases requiring an emergency department visit was taken from a US study [40]. The proportion of RSV-LRTD cases requiring hospitalization was taken from a US study [40], in the absence of Italian or European data, and the proportion admitted to intensive care was based on a study in Norway [41]. In the absence of further data available, antibiotic use was taken from a study of patients with human metapneumovirus in Italy [42].

Unit costs for healthcare resource use in Italy were obtained from published studies and Ministero della Salute data [43,44], using influenza or pneumonia as a proxy where RSV-specific data were not available, and are summarized in Supplementary Table S3. The attributable economic mean costs of RSV-LRTD cases requiring an emergency department visit and of antibiotic home treatment per episode were derived from two Italian studies on the costs of influenza and pneumonia management, respectively [45,46]. Costs related to hospitalized RSV-LRTD cases were based on mean total costs for diagnosis-related group (DRG) 79, DRG 80, DRG 089, DRG 090, DRG 092, DRG 093, DRG 096, and DRG 097 [43,44], while the costs of RSV-LRTD cases admitted to intensive care were derived from an Italian study on RSV in the pediatric population and used as a proxy due to a lack of evidence in the adult population [47]. The base case assumed that the duration of a visit to intensive care was one day. The unit costs were multiplied by resource use to calculate the average healthcare resource use costs per case in the model (Supplementary Table S3). All costs used in the model were adjusted for inflation to 2022 EUR (€) using the Italian consumer price indices from the ISTAT Rivaluta database [48].

#### 2.3.4. Demographic and Coverage Data—HR Population Aged ≥ 60 y

The model also considered the HR population aged ≥ 60 y in Italy (defined as individuals with comorbidities including respiratory, circulatory, hepatic, or renal comorbidities, or diabetes, as well as those with COPD, asthma, congestive heart failure, or weakened immunity).

For the HR population aged ≥60 y, the general Italian population data were multiplied by the proportion of individuals with respiratory, circulatory, hepatic, or renal comorbidities or diabetes, obtained from the Italian Istituto Superiore di Sanità [49]. Data on the population size used in the model are shown in Supplementary Table S1.

In the HR population aged ≥60 y, the scenarios for vaccine coverage were also derived from influenza vaccination rates, used as a proxy:

13.3%, which is the influenza vaccination rate in the 2022–2023 season in adults aged 45–64 years [50];

75%, which is the minimum target for influenza vaccination in Italy [34];

95%, which is the optimum target for influenza vaccination in Italy [34].

#### 2.3.5. Epidemiology Data— HR Population Aged ≥60 y

The mortality rate for RSV-LRTD was adjusted for the HR population aged ≥60 y based on the estimates of hospitalization risk (described in the next section).

All other epidemiology data for the HR population aged ≥60 y were assumed to be the same as for the population aged ≥75 y, due to a lack of available data (Supplementary Table S3).

#### 2.3.6. Healthcare Resource Utilization and Cost Data—HR Population Aged ≥ 60 y

Separate hospitalization risk data were used for the HR population aged ≥60 y, reflecting their higher risk of adverse outcomes compared with the general population. Two studies reported relevant data on RSV hospitalization rates among adults with comorbidities [51,52]. No Italian-specific data were available, and these were the only two available studies identified that reported data on the increase in hospitalization risk among the HR population aged ≥60 y. Therefore, results are reported for scenarios using each of these publications as a source for the input data, reflecting a conservative scenario [52] and an alternative scenario [51]. The relative increase in the hospitalization rate for HR patients compared with the general population was calculated from each study and applied to the hospitalization rate used for the general population in the base case of the model. These high-risk hospitalization rates were then applied to the RSV in-hospital mortality for patients hospitalized with RSV-LRTD in order to calculate mortality input data for the HR population aged ≥60 y. These data are summarized in Supplementary Table S4.

All other cost and healthcare resource utilization data for the HR population aged ≥60 y were assumed to be the same as for the population aged ≥75 y, due to a lack of available data.

#### 2.3.7. Vaccine Efficacy and Waning Data

Vaccine efficacy inputs for the adjuvanted RSVPreF3 OA vaccine used in the model comprised a peak vaccine efficacy value and a monthly waning value (the percentage point decrease in vaccine efficacy each month), with separate inputs for RSV-ARI and RSV-LRTD. These values were calculated based on data over two full seasons (18 months of observed data) from the AReSVi-006 phase III clinical trial, with weighted linear regression models fitted on the trial data to extrapolate beyond the clinical trial follow-up period [29].

**Figure 3:**
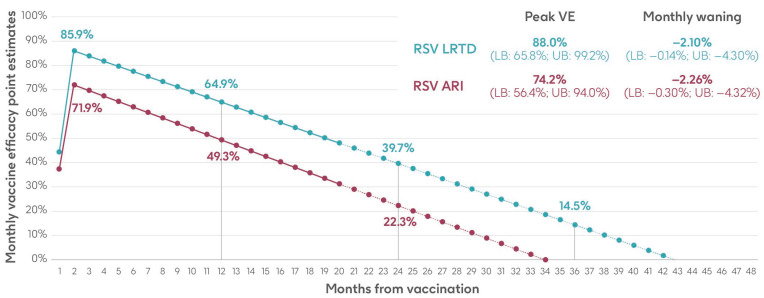
Peak vaccine efficacy and waning inputs for RSV-ARI and RSV-LRTD as used in the model (adapted from Molnar et al. 2024 [29] under a Creative Commons license http://creativecommons.org/licenses/by-nc/4.0/, accessed on 1 March 2024). ARI, acute respiratory infection; LB, lower bound; LRTD, lower respiratory tract disease; RSV, respiratory syncytial virus; UB, upper bound; VE, vaccine efficacy.

The analysis used monthly data on the number of individuals, number of RSV-ARI and RSV-LRTD cases, and follow-up time in each arm of the trial (placebo and adjuvanted RSVPreF3 OA vaccine). Monthly data were aggregated until there were at least eight cases in the placebo arm and a weighted average calculated based on the number of cases in the placebo group to give more weight to months with more robust data. Vaccine efficacy was calculated for RSV-ARI and RSV-LRTD at each time point, followed by a weighted least squares regression on both endpoints. The intercepts of the regressions provided the peak efficacy input data, and the slopes provided the monthly waning data used in the model [29]. Results are shown in Figure 3 and Supplementary Table S5.

### 2.4. Sensitivity Analysis

A univariate deterministic sensitivity analysis (UDSA) was conducted to test the effects of varying key parameter values on the projected number of RSV-ARI cases avoided across all age groups by vaccinating with the adjuvanted RSVPreF3 OA vaccine, compared with no vaccination. For vaccine efficacy and waning, the upper and lower values used in the sensitivity analysis were the upper and lower bounds calculated for the input data (Supplementary Table S5). For other parameters, values were varied by 20% above or below the base case, with values for all ages varied together.

For the HR population aged ≥60 y, two UDSAs were conducted using the hospitalization input data from Fleming et al. 2015 [52] and Osei-Yeboah et al. 2024 [51], respectively.

## 3. Results

### 3.1. Population Aged ≥ 75 y

**Figure 4:**
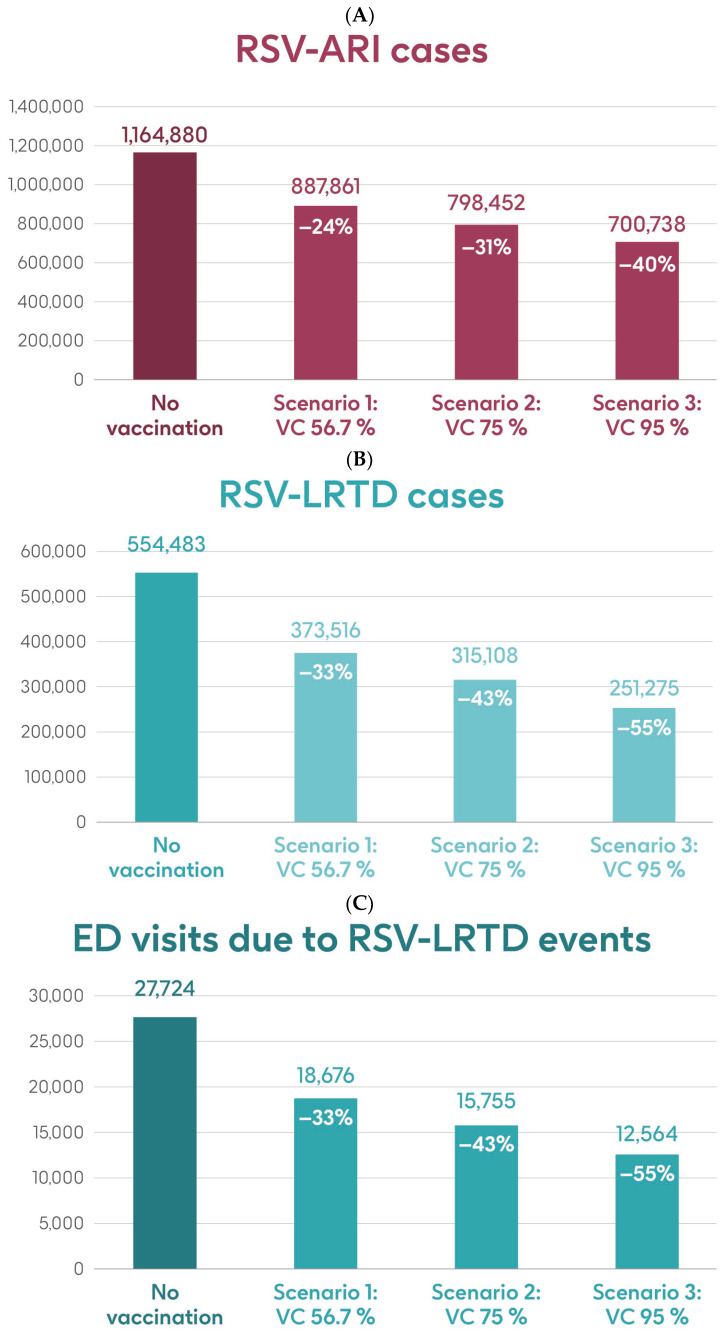

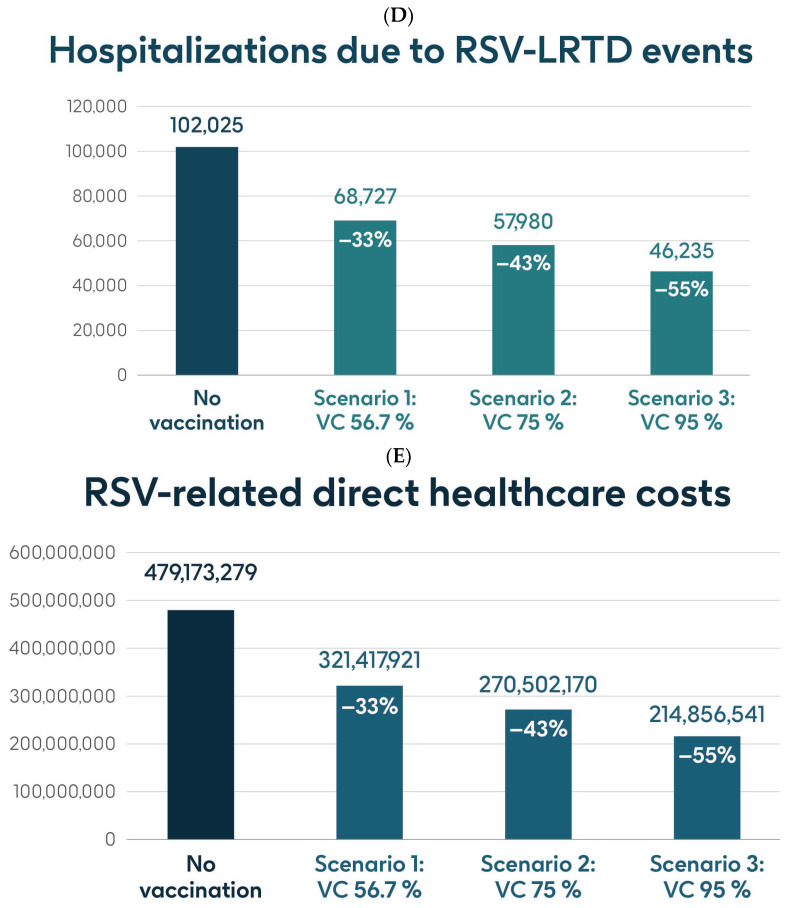
Projected numbers of (**A**) RSV-ARI cases, (**B**) RSV-LRTD cases, (**C**) RSV-LRTD ED visits, (**D**) RSV-LRTD hospitalizations, and (**E**) RSV-related direct healthcare costs for people aged ≥ 75 y in Italy over a 3-year time period with no vaccination and with vaccination using the adjuvanted RSVPreF3 OA vaccine with three scenarios for vaccine coverage. ARI, acute respiratory infection; ED, emergency department; LRTD, lower respiratory tract disease; RSV, respiratory syncytial virus; RSVPreF3 OA, RSV preFusion protein 3 older adult; VC, vaccine coverage; y, years.

Figure 4 shows the estimated number of RSV-ARI cases, RSV-LRTD cases, RSV-LRTD hospitalizations, and RSV-related direct healthcare costs in the case of no vaccination. It also shows the estimated numbers of these outcomes for coverage rates of 56.7%, 75%, and 95% for vaccination with the adjuvanted RSVPreF3 OA vaccine over a 3-year time horizon, corresponding to three consecutive RSV seasons, in individuals aged ≥75 y in Italy. Vaccination with 56.7% coverage would be expected to result in a reduction of 24% in RSV-ARI cases and a reduction of 33% in RSV-LRTD cases, hospitalizations, and direct healthcare costs (Figure 4). The projected reductions would be larger with higher coverage rates (Figure 4).

Table 1 shows the estimated number of RSV-ARI cases, RSV-LRTD cases, outpatient visits, antibiotic use, and hospitalizations related to RSV-LRTD events and RSV deaths, as well as direct healthcare costs related to RSV events with no vaccination over a 3-year time horizon in the population aged ≥75 y. It also shows the estimated number of each outcome avoided and the estimated direct healthcare costs avoided by vaccinating with the adjuvanted RSVPreF3 OA vaccine, compared with no vaccination, in each of the three coverage scenarios. In the base case, with 56.7% coverage, vaccination would be expected to reduce the number of RSV-ARI cases by 24% (277,019 avoided cases) and of RSV-LRTD cases by 33% (180,967 avoided cases). Vaccination would also reduce the number of RSV-LRTD-associated complications by 33%, including 33,298 RSV-related hospitalizations, 5394 RSV-related intensive care unit visits, and 5350 RSV-related deaths, with an estimated reduction of EUR 157,755,358 in direct healthcare costs. The number of events avoided and the estimated cost reductions would be larger with higher coverage rates (Table 1).

### 3.2. HR Population Aged ≥ 60 y

**Figure 5:**
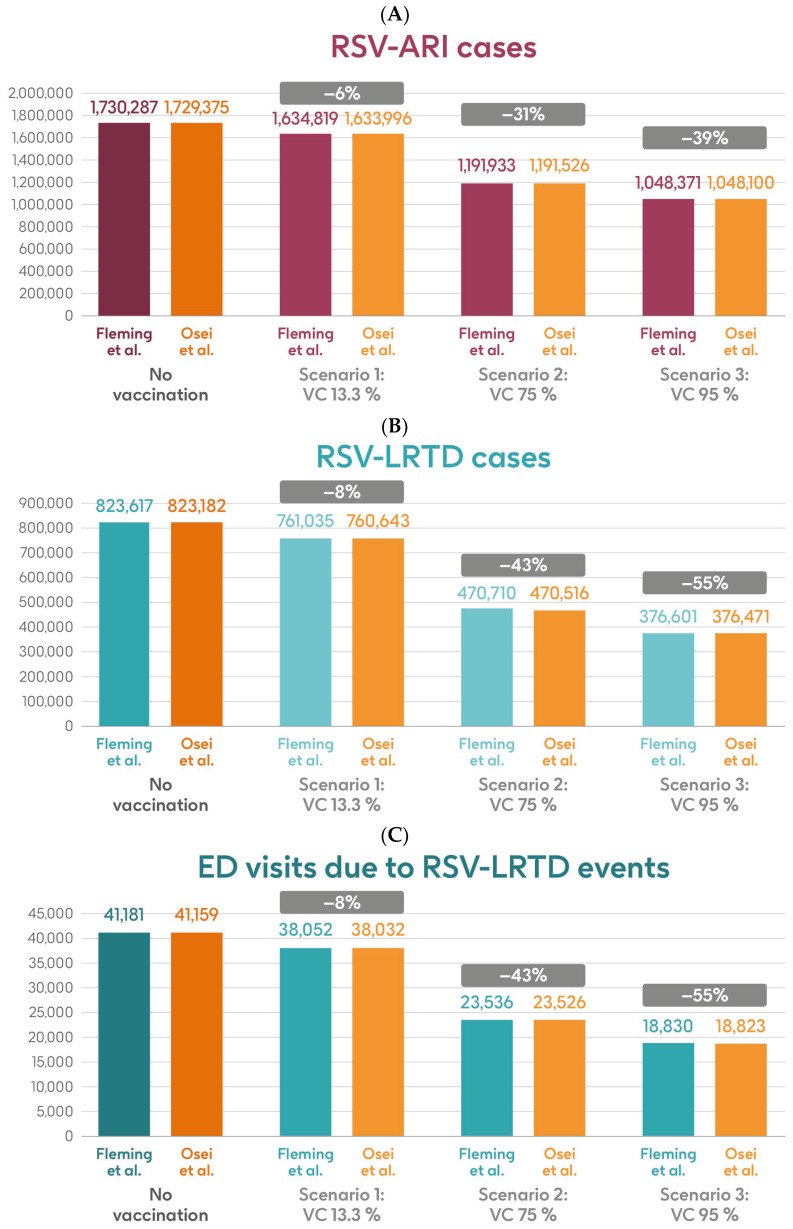

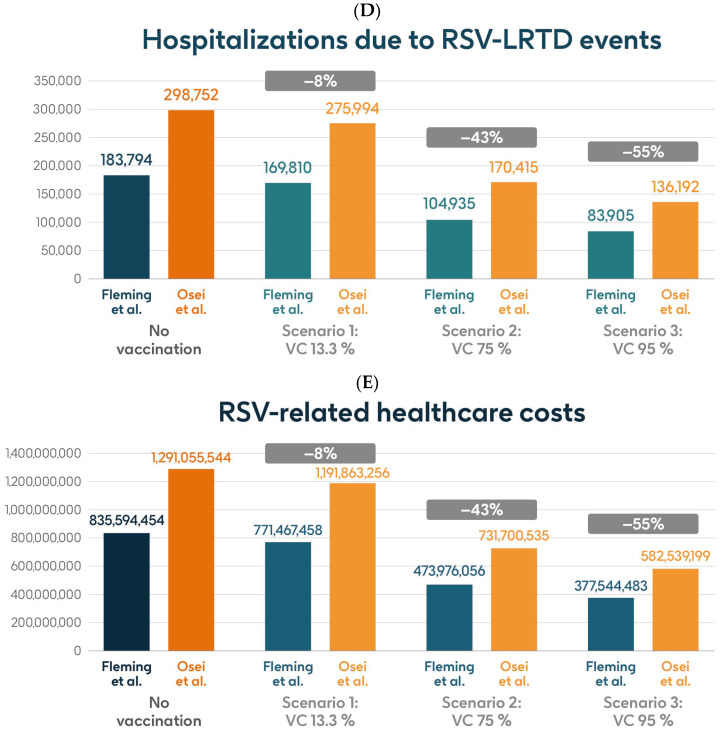
Projected numbers of (**A**) RSV-ARI cases, (**B**) RSV-LRTD cases, (**C**) RSV-LRTD ED visits, (**D**) RSV-LRTD hospitalizations, and (**E**) RSV-related direct healthcare costs for HR population aged ≥60 y in Italy over a 3-year time period with no vaccination and with vaccination using the adjuvanted RSVPreF3 OA vaccine with three scenarios for vaccine coverage. Hospitalization risk data inputs from Fleming et al. 2015 [52] or Osei-Yeboah et al. 2024 [51]. ARI, acute respiratory infection; ED, emergency department; HR, high risk; LRTD, lower respiratory tract disease; RSV, respiratory syncytial virus; RSVPreF3 OA, RSV preFusion protein 3 older adult; VC, vaccine coverage; y, years.

Figure 5 shows the estimated number of RSV-ARI cases, RSV-LRTD cases, RSV-LRTD hospitalizations, and RSV-related direct healthcare costs without vaccination and with coverage rates of 13.3%, 75%, and 95% for vaccination with the adjuvanted RSVPreF3 OA vaccine over a 3-year time horizon, corresponding to three consecutive RSV seasons, in the HR population aged ≥60 y in Italy, using hospitalization input data from Fleming et al. 2015 [52] or Osei-Yeboah et al. 2024 [51]. With a 75% coverage rate, vaccination would be expected to result in a reduction of 31% in RSV-ARI cases, and reductions of 43% in RSV-LRTD cases, hospitalizations, and direct healthcare costs.

Table 2 shows the estimated number of RSV-ARI cases, RSV-LRTD cases, outpatient visits, antibiotic use, and hospitalizations related to RSV-LRTD events, RSV deaths, and direct healthcare costs related to RSV events with no vaccination over a 3-year time horizon in the HR population aged ≥60 y in Italy, with both sources of hospitalization input data. It also shows the estimated numbers of each outcome avoided and the estimated direct healthcare costs avoided by vaccinating with the adjuvanted RSVPreF3 OA vaccine, compared with no vaccination, in each of the three coverage scenarios and with both hospitalization input data sources. In the base case, with 13.3% coverage, vaccination would be expected to reduce the number of RSV-ARI cases by 6% (95,468 avoided cases) and of RSV-LRTD cases by 8% (62,582 avoided cases). Vaccination would also reduce the number of RSV-LRTD-associated complications by 8%, including 13,984 RSV-related hospitalizations, 2265 RSV-related intensive care unit visits, and 1703 RSV-related deaths, with an estimated reduction of EUR 64,126,996 in direct healthcare costs using hospitalization risk data from Fleming et al. 2015 [52]. Using hospitalization risk data from Osei-Yeboah et al. 2024 [51], vaccination at 13.3% coverage would be expected to avoid 95,379 RSV-ARI cases (6% reduction), 62,539 RSV-LRTD cases (8% reduction), 22,758 RSV-related hospitalizations, 3687 RSV-related intensive care unit visits, and 2952 RSV-related deaths, with an estimated reduction of EUR 99,192,288 in direct healthcare costs. The number of events avoided and the estimated cost reductions would be larger with higher vaccine coverage rates, regardless of the hospitalization risk data used, with 95% coverage projected to avoid 681,916 or 681,275 RSV-ARI cases (39% reduction), 447,016 or 446,711 RSV-LRTD cases (55% reduction), 99,889 or 162,560 RSV-related hospitalizations, 16,182 or 26,335 RSV-related intensive care unit visits, and 12,181 or 21,083 RSV-related deaths, with an estimated reduction of EUR 458,049,971 or EUR 708,516,345 in direct healthcare costs (Table 2).

### 3.3. Number Needed to Vaccinate (NNV)

**Figure 6:**
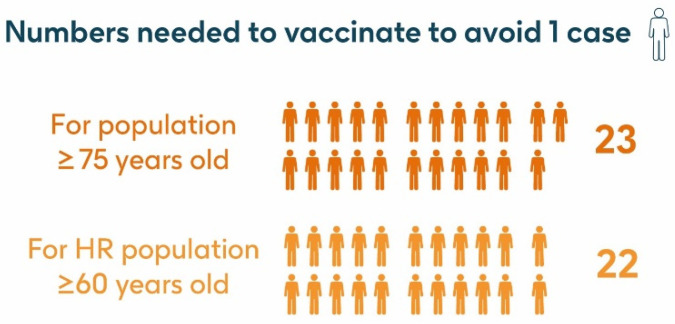
Number needed to vaccinate to avoid 1 RSV-LRTD event. HR, high risk; LRTD, lower respiratory tract disease; RSV, respiratory syncytial virus.

The number needed to vaccinate (NNV) with a single dose of the adjuvanted RSVPreF3 OA vaccine to prevent one RSV-LRTD event in the population aged ≥75 y was 23 (Figure 6). To prevent one RSV-ARI case, the NNV was 15; to prevent one RSV-LRTD hospitalization, the NNV was 124; to prevent one RSV-related intensive care unit visit, the NNV was 766; and to prevent one RSV-related death, the NNV was 772.

The NNV with a single dose of the adjuvanted RSVPreF3 OA vaccine to prevent one RSV-LRTD event in the HR population aged ≥60 y was 22 (Figure 6), and to prevent one RSV-ARI case, the NNV was 15. Using hospitalization risk data from Fleming et al. 2015 [52], the NNV to prevent one RSV-LRTD hospitalization was 102; to prevent one RSV-related intensive care unit visit, the NNV was 631; and to prevent one RSV-related death, the NNV was 890. Using hospitalization risk data from Osei-Yeboah et al. 2024 [51], to prevent one RSV-LRTD hospitalization, the NNV was 64; to prevent one RSV-related intensive care unit visit, the NNV was 397; and to prevent one RSV-related death, the NNV was 524.

### 3.4. Sensitivity Analysis

**Figure 7:**
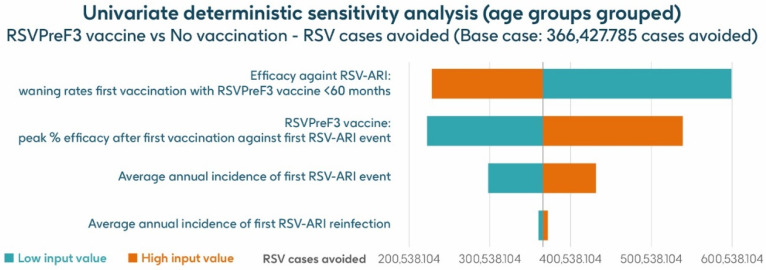
Univariate deterministic sensitivity analysis results for the projected number of RSV-ARI cases avoided by vaccinating with the adjuvanted RSVPreF3 OA vaccine in people aged ≥ 75 y in Italy over a 3-year time period with a coverage of 75%. ARI, acute respiratory infection; RSV, respiratory syncytial virus; RSVPreF3 OA, RSV preFusion protein 3 older adult; y, years.

Figure 7 shows the results of the UDSA for symptomatic RSV-ARI cases avoided by vaccination with the adjuvanted RSVPreF3 OA vaccine in individuals aged ≥75 y, with the four parameters with the greatest influence on RSV-ARI cases avoided presented as a tornado diagram. The inputs for waning rates and peak vaccine efficacy had the largest effects on the results. With lower and upper value assumptions for waning rates and peak vaccine efficacy, respectively, the model estimated approximately 599,522–228,801 and 222,820–539,378 fewer RSV-ARI cases for vaccination with the adjuvanted RSVPreF3 OA vaccine over the 3-year time horizon, compared with no vaccination. With assumptions for the low and high incidence values of symptomatic RSV-ARI (varied by 20% above or below the base case), the adjuvanted RSVPreF3 OA vaccine was estimated to avoid 298,422–431,880 RSV-ARI cases over the 3-year time period.

**Figure 8:**
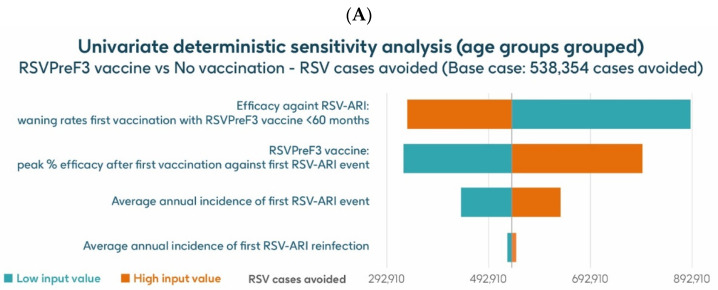

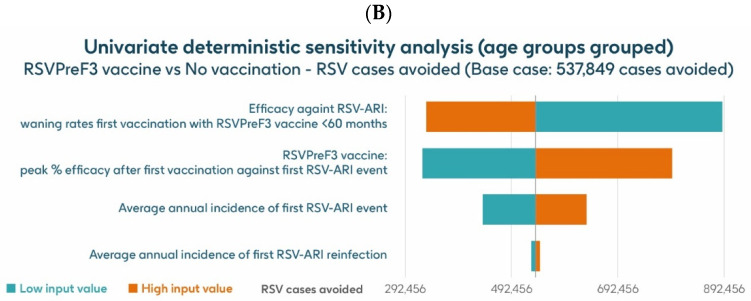
Univariate deterministic sensitivity analysis results for the projected number of RSV-ARI cases avoided by vaccinating with the adjuvanted RSVPreF3 OA vaccine in HR population aged ≥60 y in Italy over a 3-year time period with a coverage of 75%, with hospitalization risk data from (**A**) Fleming et al. 2015 [52] and (**B**) Osei-Yeboah et al. 2024 [51]. ARI, acute respiratory infection; HR, high risk; RSV, respiratory syncytial virus; RSVPreF3 OA, RSV preFusion protein 3 older adult; y, years.

The UDSA results for the number of symptomatic RSV-ARI cases avoided in the HR population aged ≥60 y are shown for the analysis using hospitalization risk data from Fleming et al. 2015 [52] in Figure 8A and for the analysis using hospitalization risk data from Osei-Yeboah et al. 2024 [51] in Figure 8B. Data for the four parameters with the largest impacts on the results are presented. In both analyses, the inputs for waning rates and peak vaccine efficacy had the largest effects on the results. For the analysis using hospitalization risk data from Fleming et al. 2015 [52], assuming the lower and upper values of waning rates and peak vaccine efficacy, respectively, the model estimated approximately 889,652–332,936 and 325,456–795,234 fewer RSV-ARI cases for vaccination with the adjuvanted RSVPreF3 OA vaccine over the 3-year time horizon, compared with no vaccination. For the analysis using hospitalization risk data from Osei-Yeboah et al. 2024 [51], the same assumptions estimated, respectively, approximately 889,146 –332,430 and 324,951–794,727 fewer RSV-ARI cases for vaccination over the 3-year period compared with no vaccination. With assumptions for the low and high incidence values of symptomatic RSV-ARI (varied by 20% above or below the base case), the adjuvanted RSVPreF3 OA vaccine was estimated to avoid 438,608–634,270 and 438,202–633,666 RSV-ARI cases over the 3 years for the analysis using hospitalization risk data from Fleming et al. 2015 [52] and from Osei-Yeboah et al. 2024 [51], respectively.

## 4. Discussion

This is the first estimate of the potential public health impact of a vaccination program with a single dose of the adjuvanted RSVPreF3 OA vaccine in OA in Italy. The adjuvanted RSVPreF3 OA vaccine provides protection against RSV for at least two consecutive seasons. The results of the present analysis indicate that vaccinating the population of adults aged ≥75 y and the HR population aged ≥60 y could potentially reduce RSV-related cases, healthcare resource use, deaths, and direct costs over a 3-year period (corresponding to three RSV seasons).

For the two populations considered in the present analysis, the projected numbers of events avoided and projected reductions in direct healthcare costs were considerably larger as the vaccine coverage rate was increased from the base case to the minimum (75%) or optimum (95%) target for influenza vaccination coverage in Italy, indicating that achieving a high coverage rate is likely to be key to the optimal implementation of a vaccine program using the adjuvanted RSVPreF3 OA vaccine in Italy. Achieving a high RSV vaccination coverage rate could reduce the number of clinical cases that often lead to the use of antibiotics and could complement national efforts to reduce antibiotic use [53]. Antibiotic prescriptions in RSV OA patients in Italy have been highlighted as common in both the inpatient [17] and the outpatient settings [54], where 63% and 61% of individuals aged 50 y and above were prescribed at least one antibiotic, respectively. The coadministration of the RSV vaccine with the seasonal influenza vaccine in these two populations may help to increase uptake by improving convenience. Involving a broad range of healthcare professionals may help to support vaccine uptake, and the World Health Organization recommends a “Health in All Policies” approach that recognizes that policies beyond the health sector can potentially affect health [55]. A life-course approach to vaccination could help to improve access to vaccines beyond infancy, including by means of diversifying vaccination pathways to make better use of existing contact points, expanding vaccine delivery to a wider range of healthcare workers and to non-healthcare settings, and strengthening the immunization of OAs, including individuals in care homes [56,57,58]. An exploration of strategies to optimize the implementation of vaccination and maximize uptake in Italy could be a valuable area for further research. Possible actions to support the achievement of adequate coverage could include building capacity on RSV prevention in healthcare professionals and interventions to improve health literacy in the general population to encourage uptake of offered RSV vaccinations.

Given the relatively recent approval of RSV vaccines [59,60,61] for use among adults aged ≥ 60 y in Europe, there are few published economic evaluations focused on the OA population. To our knowledge, this is the only public health impact analysis of RSV vaccination conducted and published in Italy. A substantial public health impact of RSV vaccination has been reported by similar studies conducted in other countries using the same static Markov model [28,29,30,62]. Consistent with our results, these studies highlight the potential of the adjuvanted RSVPreF3 OA vaccine to substantially reduce RSV disease burden among adults aged ≥ 60 y in the US [29], in Japan [30], and in Germany [62]. Other studies conducted in Europe have shown that RSV vaccination would be expected to result in not only substantial reductions in symptomatic RSV-ARI cases and RSV disease-related hospitalizations but also savings in direct medical costs [63,64]. However, comparisons with these studies are difficult, since models and assumptions (such as model structure, target population, setting, time horizon, perspective, and data input) vary from study to study. 

For example, Postma et al. [63] have used a static, cohort-based decision tree model to estimate the public health and economic impact of vaccination against RSV, and not of a specific RSV vaccine, in Belgians aged ≥60 y compared with no vaccination and from the Belgian healthcare payer perspective. Furthermore, the vaccination impact was simulated based on a generic RSV vaccine efficacy profile, drawing from published efficacy data from the RSV vaccines evaluated in adults aged 60 y in late-stage development, and the time horizon considered reflecting the vaccine duration of protection, varying from one to five years. The results of this study showed that an RSV vaccine with a 3-year duration of protection would prevent 154,728 symptomatic RSV-ARI cases over three years. Using the same symptomatic RSV-ARI incidence rate by Korsten et al. [35], and similar vaccination coverage rates, our model predicted that vaccinating Italian adults aged ≥75 y and the HR population aged ≥60 y with the adjuvanted RSVPreF3 OA would prevent 277,019 and 95,379–95,468 RSV-ARI events over a 3-year period, respectively. Differences in results could be explained by different assumptions for RSV vaccine efficacy, as also highlighted by the sensitivity analyses we conducted, which indicated that the inputs for waning rates and peak vaccine efficacy have the largest effects on the results.

Instead, Zeevat et al. [64] proposed a static decision tree model to estimate the public health and economic impact of vaccination against RSV in older adults aged ≥ 60 years for the Netherlands and ≥65 years for the United Kingdom (UK) compared with no vaccination. Furthermore, the model assumes that RSV infection is acquired once and that the time horizon is 1 year; vaccine efficacy was based on the efficacy of influenza vaccination in older adults, as well as data from a phase 3 clinical trial for maternal RSV vaccination. The results of Zeevat et al. [64] indicated that vaccinating the 4.3 million people aged ≥ 60 y in the Netherlands with the hypothetical RSV vaccine could potentially prevent 31,628 symptomatic RSV infections, 8827 GP visits, 608 hospitalizations, and 309 deaths. For vaccination of the 11.8 million people aged ≥ 65 y in the UK, the corresponding results were the prevention of 238,930 symptomatic RSV cases, 65,235 GP visits, 6277 hospitalizations, and 4320 deaths. Comparisons with the analysis developed by Zeevat et al. [64] are difficult because of differences in model types and assumptions, time horizon, epidemiological data input, and vaccine peak efficacy and waning rates. Indeed, for our analyses, a static multi-cohort Markov model was used, a 3-year time horizon was applied, reflecting observed efficacy in the AReSVi-006 phase III clinical trial [20,21], and reinfections in subsequent seasons were considered, since infection with RSV does not result in persistent immunity. Zeevat et al. [64], in the absence of data, have based the vaccine efficacy on the effectiveness of influenza vaccination in older adults, as well as data from a phase III clinical trial for RSV maternal vaccination. In the base case, a vaccine efficacy of 40% in preventing medically significant RSV lower respiratory tract infection (LRTI) and of 60% in preventing RSV LRTI with severe hypoxemia were assumed [64], while in our analyses, we used observed vaccine efficacy after a single dose of adjuvanted RSVPreF3 OA over one season (82.6% against RSV-LRTD) [20] and two seasons (67.2% against RSV-LRTD) [21]. The sensitivity analyses we conducted indicate that the inputs for waning rates and peak vaccine efficacy have the largest effects on the results, and this is aligned with Zeevat et al.’s conclusion that RSV incidence and vaccine efficacy have a significant impact on the analyses of RSV vaccination of OAs in the Netherlands and the UK [64].

RSV vaccination with the adjuvanted RSVPreF3 OA vaccine has been recognized by National Immunization Technical Advisory Groups (NITAGs), such as ACIP (US) [65] and STIKO (Germany) [66], as likely to be a cost-effective approach in the prevention of RSV in adults at risk for severe outcomes. Considering the significant social and health impacts of RSV infections in the most fragile population and the associated unmet needs, and that RSV vaccination has the potential to significantly reduce the disease burden associated with RSV in OAs, various health authorities and scientific societies have approved and recommended RSV vaccinations in several countries, including the US, UK, France, and Germany [65,66,67,68].

Uncertainties of RSV burden reported in the literature were considered in the present study and a UDSA was conducted to examine the impact of using different input values on the estimated number of symptomatic RSV-ARI cases avoided with the adjuvanted RSVPreF3 vaccine. The model’s results were robust to a series of uncertainties in key parameters used and, across all sensitivity analyses conducted, they were most sensitive to waning rates and peak vaccine efficacy, followed by annual RSV-ARI incidence. The greater impact of waning rates and peak vaccine efficacy was quite predictable since, in general, the impact of vaccination on public health is closely linked to vaccine efficacy. However, even when assuming low and high values for these key parameters, the adjuvanted RSVPreF3 vaccine was projected to have a considerable public health impact over 3 years.

This study has a number of limitations. First, the under-diagnosis of RSV infection is well established [18], and, therefore, the published incidence data used to populate the model may have led to an underestimation of the true burden of disease. Future studies adjusting RSV incidence and healthcare resource use by considering RSV under-ascertainment may better reflect the potential public health impact of RSV vaccination in Italy. Second, the incidence data available and used in the model were not stratified by age and, therefore, the same incidence rate was applied to all age groups considered. This may not reflect the real situation if we consider that susceptibility to RSV infection varies with age. Therefore, more detailed data on the incidence of RSV infection would be valuable for future analyses. Third, due to a scarcity of Italian specific data, data from other countries, other pathogens, or other age groups were used to populate the model. Obtaining more detailed Italian-specific data on RSV epidemiology and burden, in both the general and high-risk populations, would allow for more specific modeling of the public health impact of RSV in Italy. Fourth, due to the static nature of the model, subjects included in the analysis could not progress from the URTD to the LRTD transition event, whereas in the real world, URTD cases can progress to LRTD and vice versa. This may have led to an underestimation of some RSV-related events in our model. Again, using a conservative approach, it was assumed in our analysis that RSV-URTD cases do not carry any additional mortality risk and that RSV-related mortality applies only to RSV-LRTD cases; it was also assumed that RSV-URTD cases are not associated with medical care and therefore do not lead to any use of healthcare resources. More data on RSV management in the Italian outpatient setting would allow the development of further analyses, also considering specific risk groups, such as cardiorespiratory conditions and COPD, who may be at a higher risk of severe respiratory complications following RSV infection [5,6,7]. Fifth, a static model such as the one used in this analysis cannot take into account the indirect effects of vaccination (herd protection). Herd protection can only be included in dynamic models, and its exclusion from this analysis may have underestimated the public health impact of vaccination with the adjuvanted RSVPreF3 OA vaccine. Furthermore, to maintain a conservative approach to the analysis, for the efficacy data of the adjuvanted RSVPreF3 OA vaccine used in the model, a maximum vaccine efficacy value and a monthly decline value (i.e., a decrease in the percentage points of vaccine efficacy each month) were considered, with separate inputs for RSV-ARI and RSV-LRTD. These values were calculated based on data from the phase III AReSVi-006 clinical trial over two consecutive seasons [21]. However, data from the AReSVi-006 trial [21] show that one dose of the adjuvanted RSVPreF3 vaccine is able to provide protection for two consecutive seasons, and previously published regression analyses indicate substantial residual protection during the third season [29,30]. Future analyses will be needed at the conclusion of the AReSVi-006 phase III clinical trial, when longer-term data will be available, to explore the public health impact of the adjuvanted RSVPreF3 OA vaccine with different time horizons, with the objective of informing RSV vaccination strategies in Italy. Finally, the aim of this analysis was to assess the public health impact of RSV vaccination with a single dose of the adjuvanted RSVPreF3 OA over 3 years from the perspective of the Italian national health service (NHS), thus, vaccine purchase and administration costs were not considered. Further analyses would be needed to evaluate the cost-effectiveness of RSV vaccination with the adjuvanted RSVPreF3 OA from both the NHS and societal perspectives in Italy. A study in the US reported that RSV was associated with substantial productivity losses in OA, estimated at nearly US$4.7 billion per year [69]. The value of vaccination can extend beyond the impacts on vaccinated individuals and direct effects on healthcare costs, encompassing broader cost offsets in the healthcare system, effects on caregivers, effects on pathogen transmission, reduction in the development of antimicrobial resistance, and macroeconomic effects [70]. Future analyses should include the impact on societies and their economic growth in order to estimate the full value of RSV vaccination programs [71,72].

## 5. Conclusions

These results indicate that a vaccination program using one dose of the adjuvanted RSVPreF3 OA vaccine in the population aged ≥75 y and the HR population aged ≥60 y would offer the potential for substantial reductions in the disease burden associated with RSV in OAs in Italy, along with potential reductions in direct costs for the Italian NHS. These modeling data may help policymakers and clinicians to make informed decisions about recommendations for, and the implementation of, RSV vaccination in Italy.

## Figures and Tables

**Table 1:** Projected public health impact of vaccination of population aged ≥75 y with the adjuvanted RSVPreF3 OA vaccine in Italy over a 3-year time period with three scenarios for vaccine coverage.

	Projected Outcomes with No Vaccination	Projected Outcomes Avoided with Vaccination Using Adjuvanted RSVPreF3 OA Vaccine		
		Vaccine Coverage, 56.7%	Vaccine Coverage, 75%	Vaccine Coverage, 95%
Population aged ≥ 75 y	7,284,301			
Population vaccinated with adjuvanted RSVPreF3 OA vaccine		4,130,199	5,463,226	6,920,086
**Outcomes over 3-year time horizon**				
RSV-ARI cases	1,164,880	277,019	366,428	464,142
RSV-LRTD cases	554,483	180,967	239,375	303,208
Outpatient visits due to RSV-LRTD events	361,113	117,857	155,895	197,467
Antibiotic use due to RSV-LRTD events	404,988	132,283	174,979	221,639
ED visits due to RSV-LRTD events	27,724	9048	11,969	15,160
Hospitalizations due to RSV-LRTD events	102,025	33,298	44,045	55,790
ICU visits due to RSV-LRTD events	16,528	5394	7135	9038
RSV deaths	16,353	5350	7077	8964
Direct healthcare costs related to RSV-LRTD events (€)	479,173,279	157,755,358	208,671,109	264,316,738

ARI, acute respiratory infection; ED, emergency department; ICU, intensive care unit; LRTD, lower respiratory tract disease; RSV, respiratory syncytial virus; RSVPreF3 OA, RSV prefusion protein 3 older adult; y, years.

**Table 2:** Projected public health impact of vaccination of HR population aged ≥60 y with the adjuvanted RSVPreF3 OA vaccine in Italy over a 3-year time period with three scenarios for vaccine coverage and two sources of input data for hospitalization risk.

	Projected Outcomes with No Vaccination	Projected Outcomes Avoided with Vaccination Using Adjuvanted RSVPreF3 OA Vaccine		
		Vaccine Coverage, 13.3%	Vaccine Coverage, 75%	Vaccine Coverage, 95%
HR population aged ≥ 60 y	10,536,683			
Population vaccinated with adjuvanted RSVPreF3 OA vaccine		1,401,379	7,902,512	10,009,849
**Outcomes over 3-year time horizon**				
**Hospitalization risk data from Fleming et al. 2015 [52]**				
RSV-ARI cases	1,730,287	95,468	538,354	681,916
RSV-LRTD cases	823,617	62,582	352,907	447,016
Outpatient visits due to RSV-LRTD events	536,389	40,757	229,834	291,124
Antibiotic use due to RSV-LRTD events	557,892	42,431	239,272	303,078
ED visits due to RSV-LRTD events	41,181	3129	17,645	22,351
Hospitalizations due to RSV-LRTD events	183,794	13,984	78,860	99,889
ICU visits due to RSV-LRTD events	29,775	2265	12,775	16,182
RSV deaths	22,278	1703	9601	12,181
Direct healthcare costs related to RSV-LRTD events (€)	835,594,454	64,126,996	361,618,398	458,049,971
**Hospitalization risk data from Osei-Yeboah et al. 2024 [51]**				
RSV-ARI cases	1,729,375	95,379	537,849	681,275
RSV-LRTD cases	823,182	62,539	352,666	446,711
Outpatient visits due to RSV-LRTD events	536,106	40,730	229,678	290,925
Antibiotic use due to RSV-LRTD events	557,575	42,400	239,096	302,855
ED visits due to RSV-LRTD events	41,159	3127	17,633	22,336
Hospitalizations due to RSV-LRTD events	298,752	22,758	128,337	162,560
ICU visits due to RSV-LRTD events	43,398	3687	20,791	26,335
RSV deaths	38,581	2952	16,645	21,083
Direct healthcare costs related to RSV-LRTD events (€)	1,291,055,544	99,192,288	559,355,009	708,516,345

ARI, acute respiratory infection; ED, emergency department; HR, high risk; ICU, intensive care unit; LRTD, lower respiratory tract disease; RSV, respiratory syncytial virus; RSVPreF3 OA, RSV preFusion protein 3 older adult; y, years.

## Data Availability

The original contributions presented in this study are included in the article/Supplementary Materials, and further inquiries can be directed to the corresponding author.

## Supplementary Materials

The following supporting information can be downloaded at: https://www.mdpi.com/article/10.3390/vaccines13030212/s1, Table S1: Population size input data, general population and high-risk (HR) population; Table S2: Input data for epidemiology, mortality and seasonality. Table S3. Input data for healthcare resource use and costs. Table S4. Hospitalization risk and mortality input data for RSV-LRTD in the population ≥60 y HR. Table S5. Peak vaccine efficacy and waning inputs used in the model (adapted from Molnar et al. 2024 [29]).

## Correction Statement

This article has been republished with a minor correction to the copyright information and Acknowledgments Statement. This change does not affect the scientific content of the article.

## Abbreviations

AIFA: Italian Medicines Agency; ARI, acute respiratory infection; ARIEL, Adult Respiratory Syncytial Virus Cost-Effectiveness Model; AS01E, Adjuvant System containing MPL, QS-21 and liposome (25 µg MPL and 25 µg QS-21); COPD, chronic obstructive pulmonary disease; HR, high risk; LRTD, lower respiratory tract disease; MPL, monophosphoryl lipid; NNV, number needed to vaccinate; OA, older adult; QS, Quillaja saponaria; RSV, respiratory syncytial virus; RSVPreF3; RSV Prefusion protein 3; UDSA, univariate deterministic sensitivity analysis; URTD, upper respiratory tract disease; US, United States; y, years.
