# Supplementary material for: The Potential Public Health Impact of the Adjuvanted Respiratory Syncytial Virus Prefusion F Protein Vaccine Among Older Adults in Italy
Source: Vaccines (Basel). 2025 Feb 20;13(3):212. doi: 10.3390/vaccines13030212 (PMC11945443; doi:10.3390/vaccines13030212)
Supplement: Supplementary file 1 [file vaccines-13-00212-s001.zip › vaccines-3383891-supplementary.pdf]

Supplementary information

Manuscript: The potential public health impact of the adjuvanted respiratory syncytial virus prefusion F protein vaccine among older adults in Italy

Table S1. Population size input data, general population and high-risk (HR) population.

Parameter		Base case value	Source
**General population size**			(1)
	Age 60–64 y	4,148,914	
	Age 65–69 y	3,589,273	
	Age 70–74 y	3,303,871	
	Age 75–79 y	2,754,857	
	Age 80–84 y	2,260,028	
	Age 85–89 y	1,427,746	
	Age 90–109 y	841,670	
**High-risk (HR) population**			(2)
Proportion of high-risk (HR)* population	Age 60–64 y	0.512	
	Age 65–69 y	0.512	
	Age 70–74 y	0.512	
	Age 75–79 y	0.647	
	Age 80–84 y	0.647	
	Age 85–89 y	0.722	
	Age 90–109 y	0.722	
HR population size	Age 60–64 y	2,124,244	
	Age 65–69 y	1,837,708	
	Age 70–74 y	1,691,582	
	Age 75–79 y	1,782,392	
	Age 80–84 y	1,462,238	
	Age 85–89 y	1,030,833	
	Age 90–109 y	607,686	

HR, high risk; RSV, respiratory syncytial virus; y, years. *HR population is defined as individuals with comorbidities including respiratory, circulatory, hepatic, or renal comorbidities, or diabetes, as well as those with COPD, asthma, congestive heart failure, or weakened immunity.

Table S2. Input data for epidemiology, mortality and seasonality.

Parameter	Base case value	Source
**Epidemiology**		
Mean annual incidence of medically attended RSV ARI per person per year, all age groups	5.67%	(3)

Proportion of RSV ARI events that are LRTD		47.6%	Based on medically attended cases from (4)
**RSV-LRTD mortality rate**			(5)
	Age 60–64 y	0.27%	
	Age 65–74 y	0.80%	
	Age 75–84 y	2.50%	
	Age 85–109 y	4.02%	
**Seasonality by month[a]**			(6)
	January	335%	
	February	139%	
	March	12%	
	April	7%	
	May	0%	
	June	0%	
	July	0%	
	August	0%	
	September	0%	
	October	30%	
	November	188%	
	December	488%	

ARI, acute respiratory infection; LRTD, lower respiratory tract disease; RSV, respiratory syncytial virus; y, years.

a. seasonality adjustment factors for symptomatic RSV-ARI infections, calculated based on the total number of RSV PCR-confirmed cases detected each month from Influnews in Lombardia Region in Italy during the season 2022-2023 [6].

Table S3. Input data for healthcare resource use and costs.

Parameter		Base case value	Source
**Healthcare resource use**			
RSV-LRTD cases that were medically attended[a]		65.13%	(3) (4)
Proportion of RSV-LRTD cases receiving antibiotics			(7)
	Age 60–64 y	63.10%	
	Age 65–79 y	63.30%	
	Age 80–109 y	79.30%	
Proportion of RSV-LRTD cases requiring an emergency department visit			(8)
	Age 60–64 y	5.00%	

	Age 65–79 y	5.00%	
	Age 80–109 y	5.00%	
Proportion of RSV-LRTD cases hospitalized			(8)
	Age 60–64 y	3.60%	
	Age 65–74 y	10.90%	
	Age 75–109 y	18.40%	
Proportion of RSV-LRTD cases admitted to intensive care			(8) (9)
	Age 60–64 y	1%	
	Age 65–74 y	2%	
	Age 75–109 y	3%	
**Unit costs per medically attended RSV-LRTD case[b,d]**			
Outpatient visit		€96.45	(4)
Antibiotic use		€84.90	(10) (4)
Emergency visit		€280.40	(11) (4)
Hospitalization		€4,210.95[c]	(12) (13) (4)
Intensive care visit		€8,484.73	(12) (13) (14)(4)

LRTD, lower respiratory tract disease; RSV, respiratory syncytial virus; y, years.

[a] All medically attended RSV-LRTD cases were assumed to require one outpatient visit.

[b] Unit cost multiplied by proportion of medically attended RSV-LRTD cases (65.13%) to derive the average management cost of a RSV-LRTD event.

[c] Based on mean total cost of diagnosis-related-group (DRG) 79, DRG 80, DRG 089, DRG 090, DRG 092, DRG093, DRG 096, and DRG 097.

[d] All costs used in the model were adjusted for inflation to 2022 EUR (€) using the Italian consumer price indices from the ISTAT Rivaluta database (15).

Table S4. Hospitalization risk and mortality input data for RSV-LRTD in the population ≥60y HR.

Age group	General population hospitalization rate (8)	Applied relative risk	HR population hospitalization rate	RSV in-hospital mortality rate (5)	HR population mortality rate
			**Fleming et al 2015 (16)**		
60-64 y	3.60%	3.40	12.24%	7.38%	0.90%
65-69 y	10.90%	2.09	22.81%	7.38%	1.68%

70-74 y	10.90%	2.09	22.81%	7.38%	1.68%
75-79 y	18.40%	1.44	26.58%	13.60%	3.61%
80-84 y	18.40%	1.44	26.58%	13.60%	3.61%
85-89 y	18.40%	1.44	26.58%	21.85%	5.81%
90-109 y	18.40%	1.44	26.58%	21.85%	5.81%
**Osei-Yeboah et al. 2024 (17)**					
60-64 y	3.60%	2.99	10.78%	7.38%	0.80%
65-69 y	10.90%	3.10	33.76%	7.38%	2.49%
70-74 y	10.90%	3.10	33.76%	7.38%	2.49%
75-79 y	18.40%	2.67	49.21%	13.60%	6.69%
80-84 y	18.40%	2.67	49.21%	13.60%	6.69%
85-89 y	18.40%	2.81	51.66%	21.85%	11.29%
90-109 y	18.40%	2.81	51.66%	21.85%	11.29%

HR, high risk; LRTD, lower respiratory tract disease; RSV, respiratory syncytial virus; y, years.

Table S5. Peak vaccine efficacy and waning inputs used in the model (adapted from Molnar et al 2024 (18)).

	**Peak vaccine efficacy**	**Lower and upper bounds[a]**	**Standard error**
RSV-ARI	74.17%	56.39 - 94.01%	9.80%
RSV-LRTD	88.02%	65.80 - 99.20%	9.14%
	**Monthly waning [b]**	**Lower and upper bounds[a]**	**Standard error**
RSV-ARI	2.26%	0.30-4.32%	1.04%
RSV-LRTD	2.10%	0.14-4.30%	1.13%

ARI, acute respiratory infection; LRTD, lower respiratory tract disease; RSV, respiratory syncytial virus.

[a] 2.5 and 97.5 percentile.

[b] Waning timepoint <24 months.

References

1. Istituto Nazionale di Statistica (Istat). Population and households. Population: Resident population on 1st January by age (2023). Available online at: https://www.istat.it/en/population-and-households?data-and-indicators (accessed 22 January 2024).
2. Istituto Superiore di Sanità. La sorveglianza Passi d'Argento (2024). Available online at: https://www.epicentro.iss.it/passi-argento/dati/croniche (accessed 21 March 2024).
3. Korsten K, Adriaenssens N, Coenen S, Butler C, Ravanfar B, Rutter H, et al. Burden of respiratory syncytial virus infection in community-dwelling older adults in Europe (RESCEU): an international prospective cohort study. *Eur Respir J* (2021) 57(4):2002688. doi: 10.1183/13993003.02688-2020.
4. Papi A, Ison MG, Langley JM, Lee DG, Leroux-Roels I, Martinon-Torres F, et al. Respiratory Syncytial Virus Prefusion F Protein Vaccine in Older Adults. *N Engl J Med* (2023) 388(7):595-608. doi: 10.1056/NEJMoa2209604.
5. Statens Serum Institut. RS-Virus dashboard, Statens Serum Institut (22 June 2022) (2022). Available online at: https://experience.arcgis.com/experience/220fef27d07d438889d651cc2e00076c/page/RS-virus/ (accessed 9 January 2024).

6. Influnews. Sorveglianza delle sindromi simil-influenzali in Lombardia. Stagione: settimana 19-2022 - settimana 09-2023. Archivio gennaio-novembre 2023 (2023). Available online at: https://www.regione.lombardia.it/wps/portal/istituzionale/HP/DettaglioServizio/servizi-e-informazioni/Enti-e-Operatori/sistema-welfare/Promozione-della-salute/informazioni-influenza-influnews/informazioni-influenza-influnews (accessed 27 February 2024).
7. Pierangeli A, Piralla A, Uceda Renteria S, Giacomel G, Lunghi G, Pagani E, et al. Multicenter epidemiological investigation and genetic characterization of respiratory syncytial virus and metapneumovirus infections in the pre-pandemic 2018-2019 season in northern and central Italy. *Clin Exp Med* (2023) 23(6):2725-37. doi: 10.1007/s10238-022-00973-3.
8. Belongia EA, King JP, Kieke BA, Pluta J, Al-Hilli A, Meece JK, et al. Clinical Features, Severity, and Incidence of RSV Illness During 12 Consecutive Seasons in a Community Cohort of Adults >/=60 Years Old. *Open Forum Infect Dis* (2018) 5(12):ofy316. doi: 10.1093/ofid/ofy316.
9. Debes S, Haug JB, de Blasio BF, Lindstrom JC, Jonassen CM, Dudman SG. Clinical Outcome of Viral Respiratory Tract Infections in Hospitalized Adults in Norway: High Degree of Inflammation and Need of Emergency Care for Cases With Respiratory Syncytial Virus. *Front Med (Lausanne)* (2022) 9:866494. doi: 10.3389/fmed.2022.866494.
10. Potena A, Simoni M, Cellini M, Cartabellotta A, Ballerin L, Piattella M, et al. Management of community-acquired pneumonia by trained family general practitioners. *Int J Tuberc Lung Dis* (2008) 12(1):19-25.
11. Dal Negro RW, Turco P, Povero M. Cost of influenza and influenza-like syndromes (I-LSs) in Italy: Results of a cross-sectional telephone survey on a representative sample of general population. *Respir Med* (2018) 141:144-9. doi: 10.1016/j.rmed.2018.07.001.
12. Ministero della Salute. Tariffari nazionali delle prestazioni del Ssn (2024). Available online at: https://www.salute.gov.it/portale/temi/p2_6.jsp?id=3662&area=programmazioneSanitariaLea&menu=vuoto (accessed 13 March 2024).
13. Ministero della Salute. Principali caratteristiche Diagnosis Related Groups (DRG) (2013). Available online at: https://www.salute.gov.it/portale/temi/p2_6.jsp?lingua=italiano&id=1349&area=ricoveriOspedalieri&menu=sistema (accessed 5 May 2023).
14. Ravasio R, Lucioni C, Chirico G. Costo-efficacia di palivizumab versus non profilassi nella prevenzione delle infezioni da VRS nei bambini pretermine, a diversa età gestazionale. *Pharmacoeconomics Ital Res Articles* (2006) 8(2):105-17.
15. Istat.it. Rivaluta. Italian Consumer Price Index for July 2024. Available online: https://rivaluta.istat.it/Rivaluta/ (accessed on 19 March 2024).
16. Fleming DM, Taylor RJ, Lustig RL, Schuck-Paim C, Haguinet F, Webb DJ, et al. Modelling estimates of the burden of Respiratory Syncytial virus infection in adults and the elderly in the United Kingdom. *BMC Infect Dis* (2015) 15:443. doi: 10.1186/s12879-015-1218-z.
17. Osei-Yeboah R, Johannesen CK, Egeskov-Cavling AM, Chen J, Lehtonen T, Fornes AU, et al. Respiratory Syncytial Virus-Associated Hospitalization in Adults With Comorbidities in 2 European Countries: A Modeling Study. *J Infect Dis* (2024) 229(Supplement_1):S70-S7. doi: 10.1093/infdis/jiad510.
18. Molnar D, La EM, Verelst F, Poston S, Graham J, Van Bellinghen LA, et al. Public Health Impact of the Adjuvanted RSVPreF3 Vaccine for Respiratory Syncytial Virus Prevention Among Older Adults in the United States. *Infect Dis Ther* (2024) 13:827-44. doi: 10.1007/s40121-024-00939-w.
